# D‐2‐hydroxyglutaric aciduria Type I: Functional analysis of *D2HGDH* missense variants

**DOI:** 10.1002/humu.23751

**Published:** 2019-04-13

**Authors:** Ana Pop, Eduard A. Struys, Erwin E. W. Jansen, Matilde R. Fernandez, Warsha A. Kanhai, Silvy J. M. van Dooren, Senay Ozturk, Justin van Oostendorp, Pascal Lennertz, Martijn Kranendijk, Marjo S. van der Knaap, K. Michael Gibson, Emile van Schaftingen, Gajja S. Salomons

**Affiliations:** ^1^ Metabolic Unit, Department of Clinical Chemistry Amsterdam University Medical Centers, Vrije Universiteit Amsterdam, Amsterdam Neuroscience, Amsterdam Gastroenterology & Metabolism Amsterdam The Netherlands; ^2^ Department of Child Neurology Emma Children's Hospital, Amsterdam University Medical Centers, Vrije Universiteit Amsterdam and Amsterdam Neuroscience Amsterdam The Netherlands; ^3^ Department of Functional Genomics Center for Neurogenomics and Cognitive Research, Amsterdam Neuroscience, Vrije Universiteit Amsterdam Amsterdam The Netherlands; ^4^ Department of Pharmacotherapy College of Pharmacy and Pharmaceutical Sciences, Washington State University Spokane Washington; ^5^ Walloon Excellence in Life Sciences and Biotechnology Brussels Belgium; ^6^ Laboratory of Biochemistry, de Duve Institute, University of Louvain Brussels Belgium; ^7^ Department of Genetic Metabolic Diseases Amsterdam University Medical Centers, University of Amsterdam, Amsterdam Neuroscience, Amsterdam Gastroenterology & Metabolism Amsterdam The Netherlands

**Keywords:** D‐2‐HGDH, D‐2‐hydroxyglutaric aciduria, functional assay, missense variants, overexpression, residual activity

## Abstract

D‐2‐hydroxyglutaric aciduria Type I (D‐2‐HGA Type I), a neurometabolic disorder with a broad clinical spectrum, is caused by recessive variants in the *D2HGDH* gene encoding D‐2‐hydroxyglutarate dehydrogenase (D‐2‐HGDH). We and others detected 42 potentially pathogenic variants in *D2HGDH* of which 31 were missense*.* We developed functional studies to investigate the effect of missense variants on D‐2‐HGDH catalytic activity. Site‐directed mutagenesis was used to introduce 31 missense variants in the pCMV5‐*D2HGDH* expression vector. The wild type and missense variants were overexpressed in HEK293 cells. D‐2‐HGDH enzyme activity was evaluated based on the conversion of [^2^H_4_]D‐2‐HG to [^2^H_4_]2‐ketoglutarate, which was subsequently converted into [^2^H_4_]L‐glutamate and the latter quantified by LC‐MS/MS. Eighteen variants resulted in almost complete ablation of D‐2‐HGDH activity and thus, should be considered pathogenic. The remaining 13 variants manifested residual activities ranging between 17% and 94% of control enzymatic activity. Our functional assay evaluating the effect of novel *D2HGDH* variants will be beneficial for the classification of missense variants and determination of pathogenicity.

## 1 INTRODUCTION

D‐2‐hydroxyglutaric aciduria (D‐2‐HGA) is a rare neurometabolic disorder with broad phenotypic heterogeneity (Kranendijk, Struys, Salomons, Van der Knaap, & Jakobs, 2012; van der Knaap, Jakobs, Hoffmann, Duran et al., 1999; van der Knaap, Jakobs, Hoffmann, Nyhan et al., 1999). Developmental delay, hypotonia, and seizures are the most frequently observed clinical signs. D‐2‐HGA Type I (MIM# 600721) is caused by recessive variants in *D2HGDH*, the gene encoding D‐2‐hydroxyglutarate dehydrogenase (D‐2‐HGDH, MIM# 609186; Kranendijk, Struys, Gibson et al., 2010; Struys, Salomons et al., 2005). D‐2‐HGDH is a mitochondrial, FAD‐binding enzyme (Achouri et al., 2004), belonging to the α‐hydroxy acid dehydrogenases subgroup of the para‐cresol methylhydroxylase flavoprotein family (Fraaije, Van Berkel, Benen, Visser, & Mattevi, 1998). D‐2‐HGA Type II (MIM# 613657) is caused by specific heterozygous gain of function variants in *IDH2*, the gene encoding isocitrate dehydrogenase 2 (MIM# 147650; Kranendijk, Struys, van Schaftingen et al., 2010). In both types, the biochemical hallmarks are increased levels of D‐2‐hydroxyglutarate (D‐2‐HG) in body fluids due to either an impaired D‐2‐HGDH enzyme activity (Type I) or overproduction of D‐2‐HG (Type II). The accumulation of 2‐hydroxyglutarate (2‐HG) is usually detected by routine urinary organic acid screening. Since these methods are incapable of separating d‐ and L‐2‐HG, a dedicated chiral‐based assay is required to establish the biochemical diagnosis of D‐2‐HGA (Struys, Jansen, Verhoeven, & Jakobs, 2004). In the past, enzyme activity tests in fibroblasts or lymphoblasts derived from patients (Kranendijk et al., 2011; Wickenhagen, Salomons, Gibson, Jakobs, & Struys, 2009) and genetic studies were used for confirmation of the diagnosis. Implementation of next generation sequencing methodologies has resulted in molecular analysis supplanting enzymology as the first‐tier testing approach.

Thus far, 32 variants in the *D2HGDH* gene have been described in patients with D‐2‐HGA (The Human Gene Mutation Database), of which 21 are missense variants (Ali Pervaiz et al., 2011; Haliloglu et al., 2009; Kranendijk, Struys, Gibson et al., 2010; Misra et al., 2005; Struys, Korman et al., 2005; Struys, Salomons et al., 2005; Struys et al., 2006). To date, only three of these variants have been characterized via overexpression studies (Struys, Korman et al., 2005, Struys, Salomons et al., 2005). Truncating variants are usually classified as pathogenic, whereas inferring the pathogenicity of missense variants is more challenging. Bioinformatic prediction tools are important for estimating the potential of missense variants to disturb protein function but do not confirm the pathogenicity, and sometimes may fail to give correct predictions (Frigeni et al., 2017). Therefore, functional studies via site‐directed mutagenesis and enzyme assay remain pivotal in the characterization of variants of unknown significance.

Here, we describe a functional assay to characterize missense variants in the *D2HGDH* gene, while simultaneously reporting the functional evaluation of 10 novel missense variants in conjunction with 21 previously reported missense variants.

## 2 MATERIALS AND METHODS

### 2.1 Variant analysis

The coding region of the *D2HGDH* gene was analyzed as described (Kranendijk, Struys, Gibson et al., 2010). All 10 exons and the adjacent splice sites were amplified and sequenced using an ABI PRISM 3130xl Genetic Analyser (Applied Biosystems, Foster City, CA) with interpretation using Mutation Surveyor (Softgenetics, State College, PA). Nucleotide numbering of the variants reflects cDNA numbering in which +1 corresponds to the A of the ATG translation initiation codon in the reference sequence (NM_152783.3).

### 2.2 Bioinformatic prediction of pathogenicity of missense variants

Pathogenicity potential of the studied variants was estimated using three prediction software tools: PolyPhen‐2 (Adzhubei et al., 2010), SIFT (Ng & Henikoff, 2003), and MutationTaster (Schwarz, Cooper, Schuelke, & Seelow, 2014) integrated in the Alamut Visual software package, version 2.9 (Interactive Biosoftware, Rouen, France). The potential effect of the variants on messenger RNA (mRNA) splicing was also evaluated using in silico splice tools: MaxEntScan, NNsplice, and Human Splicing Finder (Alamut Visual software, version 2.9). To study sequence conservation, human and 10 homologous D‐2‐HGDH protein sequences were retrieved from the Universal Protein Knowledgebase (UniProt) and aligned using the Clustal Omega program (Sievers et al., 2011). Identical and/or similar amino acids were highlighted using BOXSHADE (https://embnet.vital‐it.ch/software/BOX_form.html).

### 2.3 Site‐directed mutagenesis

The pCMV5‐*D2HGDH* wild type construct was used for site‐directed mutagenesis and overexpression (Achouri et al., 2004). Variants were introduced in the *D2HGDH* open reading frame using specific primers (Table S1; Betsalel et al., 2012). Successful mutagenesis (as well as the absence of PCR artifacts) was confirmed by full‐length sequencing of *D2HGDH*.

### 2.4 Overexpression of missense variants in HEK293 cells

Transient expression of constructs was performed in HEK293 cells cultured in Dulbecco's Modified Eagle Medium (Thermo Fisher Scientific, Waltham, MA) supplemented with 10% heat‐inactivated fetal bovine serum, 1% penicillin/streptomycin, and 2 mM l‐glutamine. Cells were plated in 60 mm dishes and transfected at 70% confluence following 24 hr of culturing. Fugene HD transfection reagent (Promega, Madison, WI) was used following the manufacturer's specifications. A ratio of 10:2 (µl Fugene HD to µg total plasmid DNA) was determined to be optimal. Every construct was cotransfected with pEGFP‐N1 vector at a ratio of 1:50. This enabled the monitoring of transfection efficiency using fluorescent microscopy. Transfections of the variant containing vectors as well as of the pCMV5‐*D2HGDH* wild type and pCMV5 mock vector were performed in triplicate. Untransfected cells (no plasmid, only Fugene HD) also served as controls. Cells were harvested by trypsinization 48 hr posttransfection, divided in aliquots and stored at −80°C until further use.

### 2.5 Detection of overexpressed D‐2‐HGDH proteins

To verify successful transfection, SDS PAGE/Western blot analysis was performed as described (Pop et al., 2018). Immunodetection of the D‐2‐HGDH protein used a rabbit polyclonal anti‐D‐2‐HGDH primary antibody (Proteintech Group Inc, Rosemont, IL), polyclonal goat anti‐rabbit immunoglobulins/HRP secondary antibody (Agilent‐Dako, Santa Clara, CA), and enhanced chemiluminescent Lumi‐Light Plus Western blot analysis substrate (Roche Applied Science, Indianapolis, IN). Images were acquired with the ChemiDoc MP Imager (Bio‐Rad, Hercules, CA). Anti‐actin (Sigma–Aldrich, St. Louis, MO) and anti‐GFP (Abcam, Cambridge, UK) antibodies were also used as controls.

### 2.6 D‐2‐HGDH enzyme activity of transfectants

D‐2‐HGDH enzyme activity was assessed as described (Wickenhagen et al., 2009), and was optimized with respect to protein concentration, reaction time, and substrate concentration. Cell pellets were resuspended in assay buffer, disrupted by sonication, and homogenates were clarified by centrifugation. The protein content of lysates was determined by Bicinchoninic acid assay (Sigma–Aldrich), and samples were adjusted to 0.14 mg/ml protein. Enzyme assays were carried out at 37°C for 20 min, using 53.7 μΜ [^2^H_4_]D‐2‐HG (total assay volume of 120 µl). The final reaction product [^2^H_4_]L‐glutamate was quantified by LC‐MS/MS. Control assays for L‐glutamate dehydrogenase activity were performed by addition of 4.2 μM [^2^H_4_]2‐KG (total assay volume of 120 µl). Activities of wild type and variants were corrected for the HEK293 endogenous D‐2‐HGDH activity by subtracting the amount of glutamate in mock transfectants.

### 2.7 Kinetic studies of mutant D‐2‐HGDH enzymes

For a subset of variants displaying high residual activities, Michaelis‐Menten kinetic parameters (V_max_/K_m_) were evaluated to assess potential K_m_ variants. This was achieved by varying the range of [^2^H_4_]D‐2‐HG substrate from 5.4 to 107.3 µM.

## 3 RESULTS

### 3.1 ***D2HGDH*** gene variant analysis

**Figure 1:**
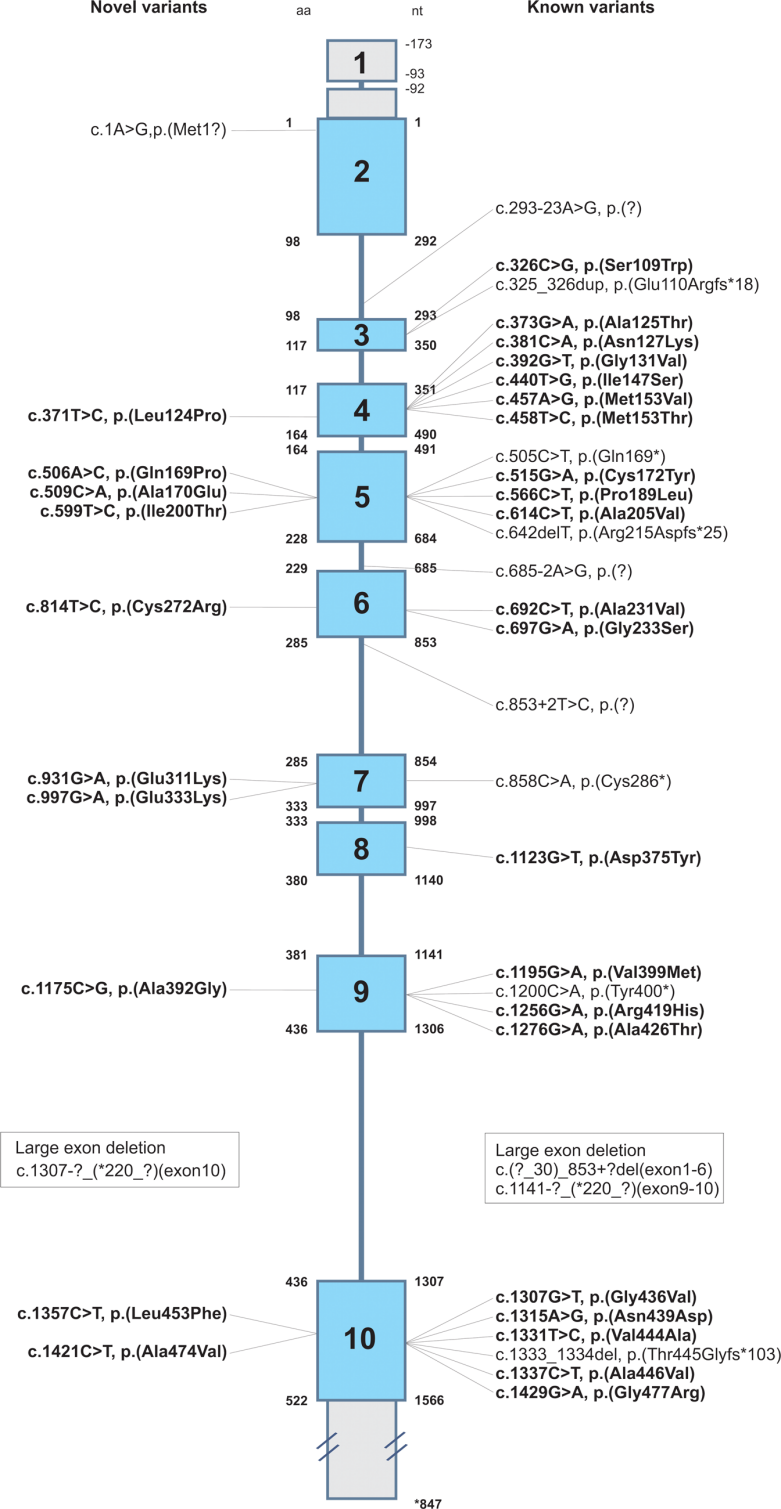
Schematic representation of the *D2HGDH* gene and distribution of all reported variants. All variants presented in this article are also included in the LOVD database (www.lovd.nl/D2HGDH). Variants were considered novel if they were not previously reported in the HGMD database and not published in the scientific literature. All missense variants are addressed in this study and are represented in bold. HGMD: Human Gene Mutation Database; LOVD: Leiden Open Variation Database

Ten novel missense variants: p.Leu124Pro, p.Gln169Pro, p.Ala170Glu, p.Ile200Thr, p.Cys272Arg, p.Glu311Lys, p.Glu333Lys, p.Ala392Gly, p.Leu453Phe, and p.Ala474Val, were detected in 10 patients suspected of D‐2‐HGDH deficiency based upon clinical and biochemical data. For two of these patients, only one heterozygous variant was detected. An overview of all previously reported and novel variants is depicted in Figure 1.

### 3.2 Functional analysis of *D2HGDH* missense variants

**Figure 2:**
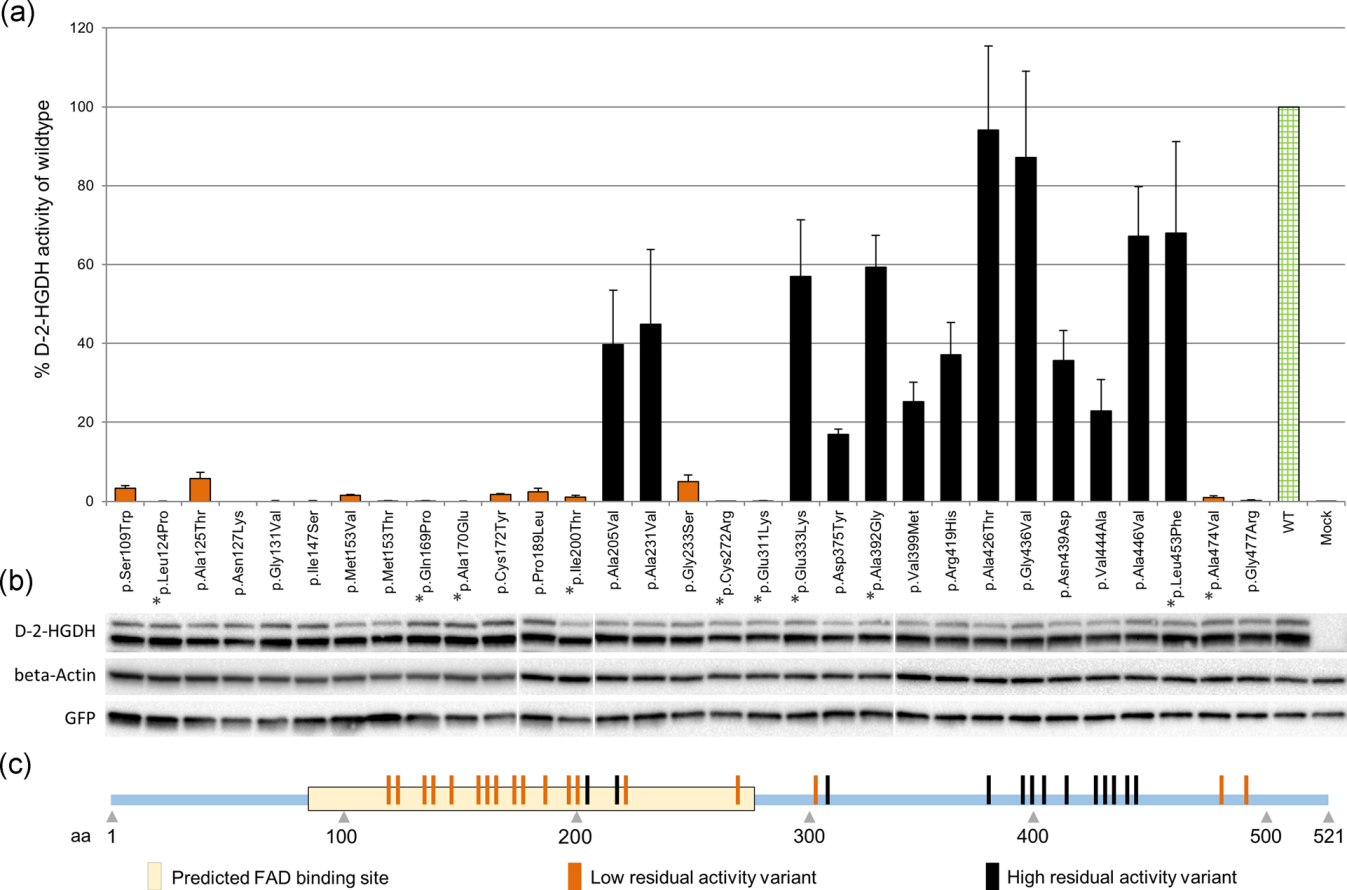
Functional analysis of *D2HGDH* missense variants transiently transfected into HEK293 cells; schematic localization of the studied variants. (a) Residual activity of the expressed alleles, shown as % of wild type transfectants; average of three independent transfections, except p.Ala392Gly (two independent transfections), and p.Ala205Val, p.Ala426Thr, and p.Asn439Asp (six independent transfections). Error bars represent standard deviation. WT stands for wild type transfectants; Mock stands for pCMV5 empty vector transfectants. Variants marked with *are novel. (b) Western blot analysis of *D2HGDH* alleles containing missense variants transfected into HEK293 using antibodies against D‐2‐HGDH, beta‐Actin, and GFP. The corresponding protein bands are shown. The white vertical lanes mark different blots. (c) Schematic representation of the D‐2‐HGDH protein and distribution of the studied missense variants. GFP: green fluorescent protein

The effect of the missense variants on the function of D‐2‐HGDH enzyme was studied by overexpression in HEK293 cells. In total, 31 missense variants were analyzed, including 10 novel variants. All variants were independently introduced in the coding region of the *D2HGDH* gene and the resulting recombinant constructs as well as the appropriate controls, were overexpressed in HEK293 cells. All transfections were performed in triplicate and average activities are displayed in Figure 2a. The wild type *D2HGDH* transfectant activity was 104 ± 16 nmol [^2^H_4_]L‐glutamate/h/mg total protein, approximately 150‐fold higher than mock transfectants. Missense variant activities are expressed as percentage of wild type activity. The majority of variants significantly impaired D‐2‐HGDH activity (<6% of wild type activity). For 13 variants, the activities varied from 17% to 94%. Western blot analysis showed protein expression for all variant proteins, confirming successful transfections (Figure 2b). Interestingly, all proteins appeared to be stable, regardless of their enzymatic activities. Most of the high residual activity D‐2‐HGDH variants presented with both altered K_m_ and V_max_ parameters, in varying degrees (Figure S1), with the exception of p.Ala426Thr, p.Leu453Phe, and p.Asn493Asp, which had close to the low normal range of V_max,_ but showed significantly increased K_m_.

It was of interest to compare the results of the overexpression studies with in silico predictions. The multiple sequence alignment of D‐2‐HGDH orthologs revealed that many missense variants mapped to relatively conserved residues (Figure S2). The pathogenicity of the 31 studied missense variants was evaluated using the PolyPhen‐2, SIFT, and Mutation Taster software tools (Alamut Visual, version 2.9; Table S2). The results were compared with each other and also against the functional studies of the missense variants in HEK293 cells. For 20 out of 31 variants (64.5%), all three software tools were unanimous in assigning pathogenicity predictions and were also in agreement with the results of the functional studies in HEK293 cells. For 11 variants (35.5%) the predicted pathogenicity was not consistent among the used software tools and the measured activities, being differently estimated either by one prediction tool (four variants, 13%), by two prediction tools (five variants, 16%) or by all (two variants, 6.5%). The potential effect of the variants on mRNA splicing was also evaluated using in silico splice tools: MaxEntScan, NNSPLICE, and Human Splicing Finder (Alamut Visual software, version 2.9). Only for c.997G>A (p.Glu333Lys) variant the predicted splicing events appeared to be significant – predicted change at donor site 1 bps downstream: −71.2% (MaxEntScan: −100.0%; NNSPLICE: −99.7%; Human Splicing Finder: −13.9%).

## 4 DISCUSSION

We developed an overexpression system for functional characterization of *D2HGDH* missense variants and analyzed the effects of a total of 31 missense variants, of which 10 were novel (Figure 2). All variants were detected in patients with increased urinary excretion of D‐2‐HG, in the range of the published D‐2‐HGA Type I patients suggesting D‐2‐HGA (Type I) at the biochemical level (Kranendijk et al., 2012).

The following variants are being classified as pathogenic variants based upon minimal residual D‐2‐HGDH activities: p.Ser109Trp, p.Leu124Pro, p.Ala125Thr, p.Asn127Lys, p.Gly131Val, p.Ile147Ser, p.Met153Val, p.Met153Thr, p.Gln169Pro, p.Ala170Glu, p.Cys172Tyr, p.Pro189Leu, p.Ile200Thr, p.Gly233Ser, p.Cys272Arg, p.Glu311Lys, p.Ala474Val, p.Gly477Arg. Thirteen variants revealed between 17% and 94% of wild‐type D‐2‐HGDH activity, making their clinical classification more challenging (Figure 2a). A summary of available information on all missense variants with substantial residual activity is presented in Table 1. Missense variants presenting the highest residual activities are briefly discussed below.

**Table 1:** Missense variants with substantial residual D‐2‐HGDH activity

No.	Variant	Enzyme activity (% of wild type)	#Patients	Format	gnomAD browser beta (% of control alleles)	Probable role of allele	Comments/Variant reference
1	p.Ala426Thr	94	3	Chet	0.7; 13 homozygous	Likely non‐pathogenic	Complete impairment of D‐2‐HGDH in 1 fibroblast line; (patient 5, Kranendijk, Struys, Gibson et al., 2010)
2	p.Gly436Val	87	1	Het	0.0004	Likely non‐pathogenic	Also SSADH deficiency; (Struys et al., 2006)
3	p.Leu453Phe	68	1	Het	0.05	Likely non‐pathogenic	Also *IDH2* variant; Type II D‐2‐HGA; (Kranendijk, Struys, van Schaftingen et al., 2010)
4	p.Ala446Val	67	1	Homoz	0.00219	Likely non‐pathogenic	Poor sequence conservation; (Kranendijk, Struys, Gibson et al., 2010)
5	p.Ala392Gly	59	1	Het	0	Pathogenicity unclear	Undetectable D‐2‐HGDH activity in patient fibroblasts; (this study)
6	p.Glu333Lys	57	1	Homoz	0	Pathogenicity unclear	Possible missplicing (variant location = 3′ end of exon 7); (this study)
7	p.Ala231Val	45	1	Homoz	0	Likely pathogenic	(Kranendijk, Struys, Gibson et al., 2010)
8	p.Ala205Val	40	1	Chet	0	Likely pathogenic	(Kranendijk, Struys, Gibson et al., 2010)
9	p.Arg419His	37	1	Homoz	0.0047	Pathogenic	Impaired enzyme activity in fibroblasts; (Kranendijk, Struys, Gibson et al., 2010))
10	p.Asn439Asp	36	1	Chet	0	Pathogenic	(Struys, Korman et al., 2005)
11	p.Val399Met	25	1	Chet	0.0012	Pathogenic	(Kranendijk, Struys, Gibson et al., 2010)
12	p.Val444Ala	23	1	Homoz	0	Pathogenic	(Struys, Salomons et al., 2005)
13	p.Asp375Tyr	17	2^a^	Chet	0.0018	Pathogenic	(Misra et al., 2005)

*Note.* Chet: compound heterozygous; D‐2‐HGDH: D‐2‐hydroxyglutarate dehydrogenase; gnomAD browser beta: genome aggregation database (http://gnomad.broadinstitute.org/); Het: heterozygous; Homoz: homozygous; IDH2: isocitrate dehydrogenase 2; SSADH: succinic semialdehyde dehydrogenase.

^a^monozygotic twins.

The p.Ala426Thr variant displays the highest activity among the studied variants (94%), similar to the wild type transfectants activity (Figure 2a). Together with the observation that this variant is extremely frequent in the population (0.7%) and has even been found in the homozygous state in 13 individuals, this suggests that p.Ala426Thr does not affect the activity and is not disease causing. However, fibroblasts derived from a patient compound heterozygote for p.Ala426Thr and p.Tyr400X manifested complete impairment of D‐2‐HGDH enzymatic activity (Table 1; patient 5 [Kranendijk, Struys, Gibson et al., 2010]). One potential explanation might be that the p.Tyr400X mutant has a kind of dominant negative effect on the p.Ala426Thr variant when they associate in dimers or heterooligomers. Curiously, this dominant negative effect would need to be specific for p.Ala426Thr variant and not be observed when the p.Tyr400X mutant associates with the wild type protein, otherwise obligate heterozygotes for the p.Tyr400X would show D‐2‐HGA. The most obvious explanation is therefore that the p.Ala426Thr allele is not associated with the inborn error D‐2‐HGA Type I and that in all three unrelated cases, the causative allele(s) remains unidentified.

Two of the variants presenting high residual activity were detected in the heterozygous state in patients with increased urinary D‐2‐HG excretion and diagnosed with other metabolic diseases. The p.Gly436Val variant (87% residual activity) was detected in a patient with succinic semialdehyde dehydrogenase (SSADH) deficiency (Struys et al., 2006), and the novel variant, p.Leu453Phe (68% residual activity), was found in a patient diagnosed with D‐2‐HGA Type II. The mild effect of these variants on D‐2‐HGDH activity and the fact that they are found in heterozygous state in patients diagnosed with other metabolic deficiencies suggests they are likely rare nonpathogenic variants (Table 1). However, we cannot exclude that the mild decrease in D‐2‐HGDH activity could also contribute to the phenotype of these SSADH or D‐2‐HGA Type II patients.

p.Ala392Gly (59% residual activity) was detected in the heterozygous state as the sole potentially pathogenic allele in one patient. Patient‐derived fibroblast analyses revealed a complete impairment of D‐2‐HGDH activity. Yet, the clinical significance of the p.Ala392Gly variant is not fully elucidated, and therefore we cannot exclude that other unidentified variants may exist.

p.Ala446Val displays 67% residual activity. Conservation of Ala446 is poor across D‐2‐HGDH orthologs (Figure S2) and the software prediction tools predict the p.Ala446Val variant as benign (Table S2). Taken together, these data suggest that p.Ala446Val is likely a nonpathogenic variant. Interestingly, this variant was reported in homozygous form in one patient biochemically presenting D‐2‐HGA.

p.Glu333Lys (57% residual activity) is a novel variant observed in the homozygous state in a single patient. The relevant nucleotide substitution (c.997G>A) resides at the distal 3′end of exon 7, potentially altering splice‐donor sites for intron 7. Splice prediction tools (Table S2) suggest erroneous splicing and mRNA studies will be required to confirm the pathogenicity of this allele.

Kinetic studies of variants demonstrating high residual D‐2‐HGDH activity revealed altered Michaelis–Menten parameters (Figure S1), which might explain the higher residual activity of some variants, detected in the overexpression system with saturating substrate concentration.

Many of the variants that severely impair D‐2‐HGDH activity cluster in proximity to the FAD‐binding region (Figure 2c), potentially revealing a direct mechanism for their severe effect on enzyme function. With the exception of two variants (p.Ala474Val and p.Gly477Arg), those clustered near the C terminal region of the D‐2‐HGDH protein revealed higher residual activities. Previous studies of the structural role of the C‐terminus in D‐2‐HGDH structure/function revealed that p.Val399Met, p.Arg419His and p.Ala446Val did not alter protein homodimerization or intracellular localization (Lin et al., 2015). Eventual crystallization and x‐ray diffraction analyses of D‐2‐HGDH will be illuminating in placing the variants we have identified within a 3‐dimensional context.

*D2HGDH* transcriptional regulation studies in colorectal cancer have recently been reported (Han et al., 2018). These investigators identified a putative Hif‐1α transcription factor binding site in the *D2HGDH* promoter, suggesting that Hif‐1α controls both the expression of D‐2‐HGDH as well as D‐2‐HG levels. Comparable promoter studies are needed in our patients for whom variants with high residual activities and/or a single potentially pathogenic allele, were identified.

The pathogenicity potential of the studied variants was also estimated using PolyPhen‐2, SIFT and MutationTaster prediction software tools (Alamut Visual software package, version 2.9; Table S2a–d). While for 64.5% of the variants the in silico predictions are in agreement with the measured activities, for 35.5% of variants there are dissimilarities within in silico predictions as well as between these and functional data, thus emphasizing the importance of the functional assays and their role in the classification of variants of unknown significance.

In conclusion, we present the functional characterization of 31 *D2HGDH* missense variants, and the identification of 10 novel variants, representing the largest study to date in this rare population. Our functional tests proved effective in classifying missense variants associated with severely impaired D‐2‐HGDH activity. However, the characterization of high residual activity missense variants is still challenging, and in these cases, our method could only provide data suggestive of further investigation. Additional studies are in progress on variants with high as well as intermediate levels, of expressed D‐2‐HGDH activities.

## Supporting information

Supporting information
