# Supplementary material for: D‐2‐hydroxyglutaric aciduria Type I: Functional analysis of D2HGDH missense variants
Source: Hum Mutat. 2019 Apr 13;40(7):975–82. doi: 10.1002/humu.23751 (PMC6619364; doi:10.1002/humu.23751)
Supplement: Supplementary file 1 — Supporting information [file HUMU-40-975-s001.pdf]

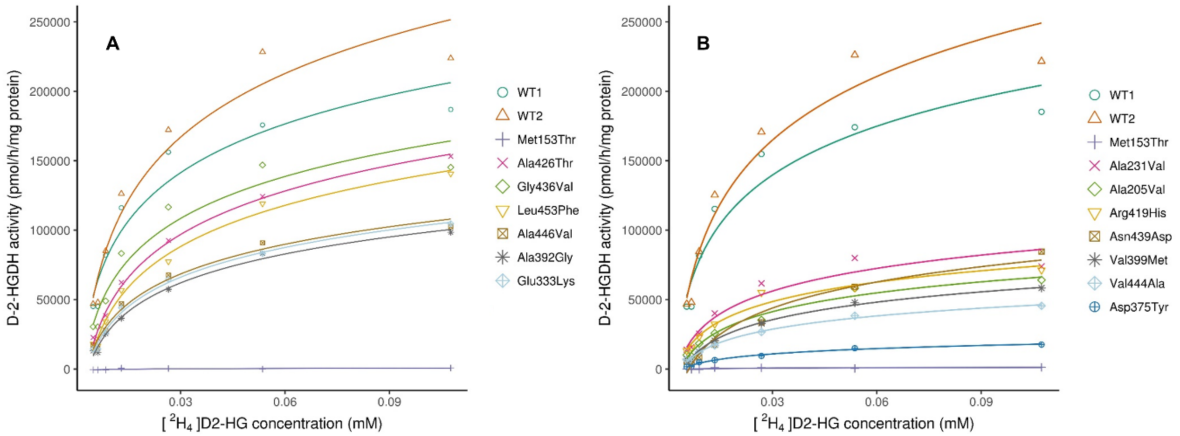

A

	Ala426Thr	Gly436Val	Leu453Phe	Ala446Val	Ala392Gly	Glu333Lys
V_{max}	205.35 ± 13.36	187.12 ± 18.92	197.89 ± 17.53	135.56 ± 11.44	138.14 ± 10.51	138.89 ± 10.80
K_m	0.036 ± 0.0053	0.021 ± 0.0054	0.041 ± 0.0079	0.030 ± 0.0060	0.040 ± 0.0066	0.030 ± 0.0062

	WT1	WT2	Met153Thr
V_{max}	224.21 ± 18.55	286.06 ± 26.22	1.65 ± 1.49
K_m	0.016 ± 0.0037	0.020 ± 0.0049	0.051 ± 0.0940

B

	Glu333Lys	Ala205Val	Arg419His	Asn439Asp	Val399Met	Val444Ala	Asp375Tyr
V_{max}	138.89 ± 10.80	89.80 ± 8.19	88.01 ± 7.25	193.05 ± 35.31	86.32 ± 7.58	63.46 ± 3.99	25.84 ± 2.88
K_m	0.030 ± 0.0062	0.037 ± 0.0075	0.023 ± 0.0048	0.130 ± 0.0370	0.050 ± 0.0087	0.039 ± 0.0054	0.045 ± 0.0110

Supp. Figure S1. **Kinetic analysis of high residual activity D-2-HGDH variants**. **A.** Variants with residual activities above 50%; **B.** Variants with residual activities between 17 and 50%.

Kinetic analyses were conducted using a range of [^{2}H4]D-2-HG substrate, from 5.4 to 107.3 µM (in a total assay volume of 120 µl). Data were analyzed using GraphPad Prism software and the Michaelis-Menten equation. V_{max} and K_m values are displayed ± Standard Error. The positive controls (WT1 and WT2) and negative control (p.Met153Thr variant, showing no residual activity) are displayed in both graphs and the V_{max} and K_m values are presented in the box.

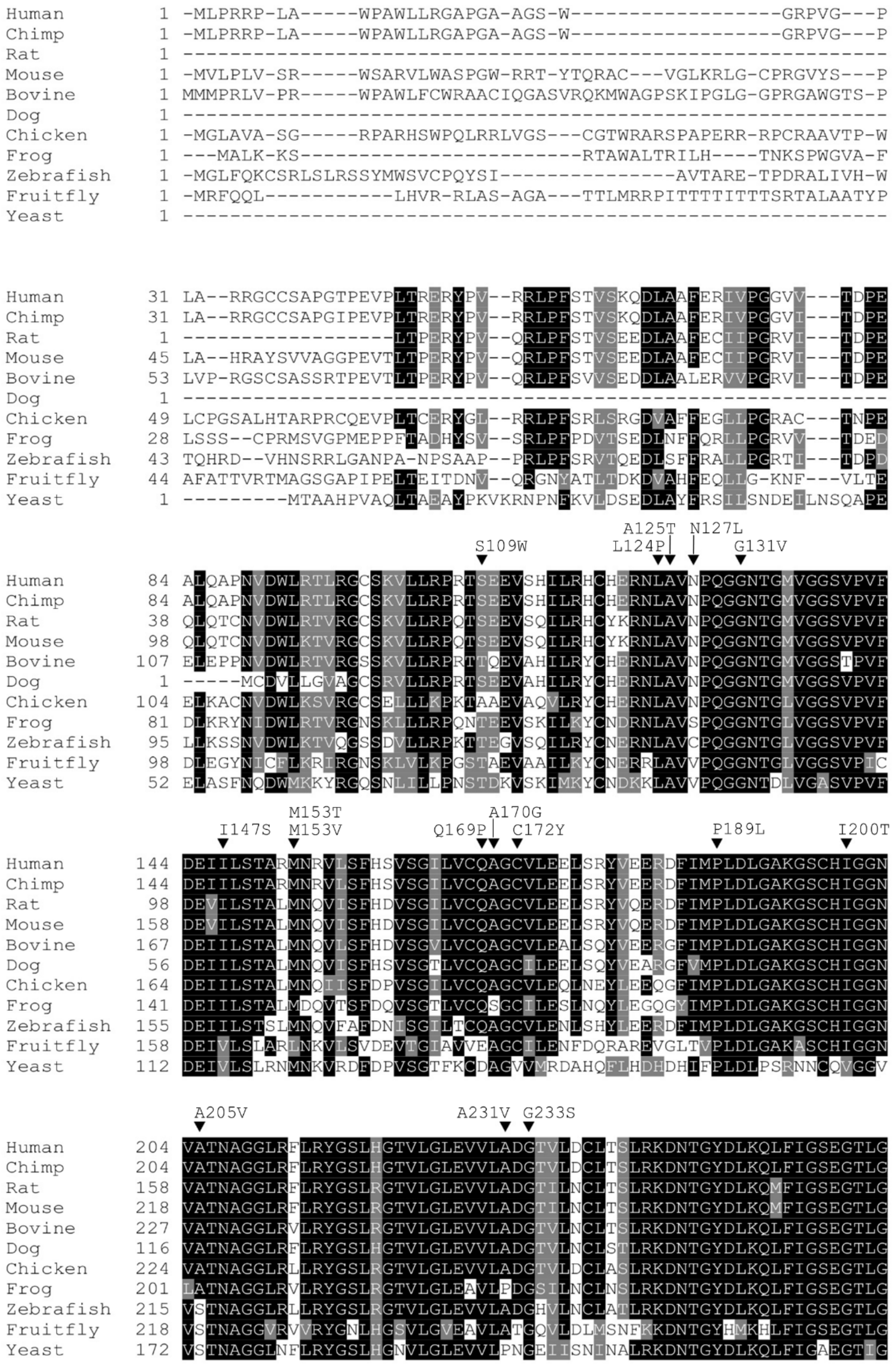

Human 1 -MLPRRP-LA-----WPAWLLRGAPGA-AGS-W------------------GRPVG---P
Chimp 1 -MLPRRP-LA-----WPAWLLRGAPGA-AGS-W------------------GRPVG---P
Rat 1 --
Mouse 1 -MVLPLV-SR-----WSARVLWASPGW-RRT-YTQRAC---VGLKRLG-CPRGVYS---P
Bovine 1 MMMPRLV-PR-----WPAWLFCWRAACIQGASVRQKMWAGPSKIPGLG-GPRGAWGTS-P
Dog 1 --
Chicken 1 -MGLAVA-SG-----RPARHSWPQLRRLVGS---CGTWRARSPAPERR-RPCRAAVTP-W
Frog 1 ---MALK-KS-------------------------RTAWALTRILH----TNKSPWGVA-F
Zebrafish 1 -MGLFQKCSRLSLRSSYMWSVCPQYSI---------------AVTARE-TPDRALIVH-W
Fruitfly 1 -MRFQQL-----------LHVR-RLAS-AGA---TTLMRRPITTTTITTTSRTALAATYP
Yeast 1 --
Human 31 LA--RRGCCSAPGTPEVPLTRERYPV--RRLPFSTVSKQDLAAFERIVPGGVV---TDPE
Chimp 31 LA--RRGCCSAPGIPEVPLTRERYPV--RRLPFSTVSKQDLAAFERIVPGGVI---TDPE
Rat 1 ------------------LTPERYPV--QRLPFSTVSEEDLAAFECIIPGRVI---TDPE
Mouse 45 LA--HRAYSVVAGGPEVTLTPERYPV--QRLPFSTVSEEDLAAFECIIPGRVI---TDPE
Bovine 53 LVP-RGSCSASSRTPEVTLTPERYPV--QRLPFSVVSEDDLAALERVVPGRVI---TDPE
Dog 1 --
Chicken 49 LCPGSALHTARPRCQEVPLTCERYGL--RRLPFSRLSRGDVAFFEGLLPGRAC---TNPE
Frog 28 LSSS--CPRMSVGPMEPPFTADHYSV--SRLPFPDVTSEDLNFFQRLLPGRVV---TDED
Zebrafish 43 TQHRD--VHNSRRLGANPA-NPSAAP--PRLPFSRVTQEDLSFFRALLPGRTI---TDPD
Fruitfly 44 AFATTVRTMAGSGAPIPELTEITDNV--QRGNYATLTDKDVAHFEQLLG-KNF---VLTE
Yeast 1 ---------MTAAHPVAQLTAEAYPKVKRNPNFKVLDSEDLAYFRSILSNDEILNSQAPE
A125T N127L
S109W L124P G131V
Human 84 ALQAPNVDWLRTLRGCSKVLLRPRTSEEVSHILRHCHERNLAVNPQGGNTGMVGGSVPVF
Chimp 84 ALQAPNVDWLRTLRGCSKVLLRPRTSEEVSHILRHCHERNLAVNPQGGNTGMVGGSVPVF
Rat 38 QLQTCNVDWLRTVRGCSKVLLRPQTSEEVSQILRHCYKRNLAVNPQGGNTGMVGGSVPVF
Mouse 98 QLQTCNVDWLKTVRGCSKVLLRPQTSEEVSQILRHCYKRNLAVNPQGGNTGMVGGSVPVF
Bovine 107 ELEPPNVDWLRTVRGSSKVLLRPRTTQEVAHILRYCHERNLAVNPQGGNTGMVGGSTPVF
Dog 1 -----MCDVLLGVAGCSRVLLRPRTSEEVAHILRYCHERNLAVNPQGGNTGMVGGSVPVF
Chicken 104 ELKACNVDWLKSVRGCSELLLKPKTAAEVAQVLRYCHERNLAVNPQGGNTGLVGGSVPVF
Frog 81 DLKRYNIDWLRTVRGNSKLLLRPQNTEEVSKILKYCNDRNLAVSPQGGNTGLVGGSVPVF
Zebrafish 95 LLKSSNVDWLKTVQGSSDVLLRPKTTEGVSQILRYCNERNLAVCPQGGNTGLVGGSVPVF
Fruitfly 98 DLEGYNICFLKRIRGNSKLVLKPGSTAEVAAILKYCNERRLAVVPQGGNTGLVGGSVPIC
Yeast 52 ELASFNQDWMKKYRGQSNLILLPNSTDKVSKIMKYCNDKKLAVVPQGGNTDLVGASVPVF
M153T A170G
I147S M153V Q169P C172Y P189L I200T
Human 144 DEIILSTARMNRVLSFHSVSGILVCQAGCVLEELSRYVEERDFIMPLDLGAKGSCHIGGN
Chimp 144 DEIILSTARMNRVLSFHSVSGILVCQAGCVLEELSRYVEERDFIMPLDLGAKGSCHIGGN
Rat 98 DEVILSTALMNQVISFHDVSGILVCQAGCVLEELSRYVQERDFIMPLDLGAKGSCHIGGN
Mouse 158 DEVILSTALMNQVISFHDVSGILVCQAGCVLEELSRYVQERDFIMPLDLGAKGSCHIGGN
Bovine 167 DEIILSTALMNQVLSFHDVSGVLVCQAGCVLEALSQYVEERGFIMPLDLGAKGSCHIGGN
Dog 56 DEIILSTALMNQVISFHSVSGTLVCQAGCILEELSQYVEARGFVMPLDLGAKGSCHIGGN
Chicken 164 DEIILSTALMNQIISFDPVSGILVCQAGCVLEQLNEYLEEQGFIMPLDLGAKGSCHIGGN
Frog 141 DEIILSTALMDQVTSFDQVSGTLVCQSGCILESLNQYLEGQGYIMPLDLGAKGSCHIGGN
Zebrafish 155 DEIILSTSLMNQVFAFDNISGILTCQAGCVLENLSHYLEERDFIMPLDLGAKGSCHIGGN
Fruitfly 158 DEIVLSLARLNKVLSVDEVTGIAVVEAGCILENFDQRAREVGLTVPLDLGAKASCHIGGN
Yeast 112 DEIVLSLRNMNKVRDFDPVSGTFKCDAGVVMRDAHQFLHDHDHIFPLDLPSRNNCQVGGV
A205V A231V G233S
Human 204 VATNAGGLRFLRYGSLHGTVLGLEVVLADGTVLDCLTSLRKDNTGYDLKQLFIGSEGTLG
Chimp 204 VATNAGGLRFLRYGSLHGTVLGLEVVLADGTVLDCLTSLRKDNTGYDLKQLFIGSEGTLG
Rat 158 VATNAGGLRFLRYGSLRGTVLGLEVVLADGTILNCLTSLRKDNTGYDLKQMFIGSEGTLG
Mouse 218 VATNAGGLRFLRYGSLRGTVLGLEVVLADGTILNCLTSLRKDNTGYDLKQMFIGSEGTLG
Bovine 227 VATNAGGLRVLRYGSLRGTVLGLEVVLADGTVLNCLTSLRKDNTGYDLKQLFIGSEGTLG
Dog 116 VATNAGGLRFLRYGSLHGTVLGLEVVLADGTVLNCLSTLRKDNTGYDLKQLFIGSEGTLG
Chicken 224 VATNAGGLRLLRYGSLRGTVLGLEVVLADGTVLDCLASLRKDNTGYDLKQLFIGSEGTLG
Frog 201 LATNAGGLRVLRYGSLRGTVLGLEAVLPDGSILNCLNSLRKDNTGYDLKQLFIGSEGTLG
Zebrafish 215 VSTNAGGLRLLRYGSLRGTVLGLEVVLADGHVLNCLATLRKDNTGYDLKQLFIGSEGTLG
Fruitfly 218 VSTNAGGVRVVRYGNLHGSVLGVEAVLATGQVLDLMSNFKKDNTGYHMKHLFIGSEGTLG
Yeast 172 VSTNAGGLNFLRYGSLHGNVLGLEVVLPNGEIISNINALRKDNTGYDLKQLFIGAEGTIG

```
                          C272R                                                 E311L
                          ▼                                                     ▼
Human      264 IITTVSILCPPKPRAVNVAFLGCPGFAEVLQTFSTCKGMLGEILSAFEFMDAVCMQLVG-
Chimp      264 IITAVSILCPPKPRAVNVAFLGCPGFAEVLQTFSTCKGMLGEILSAFEFMDAVCMQLVG-
Rat        218 VITAVSIVCPPRPKAVNVAFLGCPGFTEVLQTFRTCKGQLGEILSAFEFMDAECMQLVG-
Mouse      278 VITAVSIVCPPRPKAVNVAFLGCPGFAEVLQTFRTCRGMLGEILSAFEFMDTECMQLVG-
Bovine     287 VITAVSILCPPKPSTVNVAFLGCPGFAEVLQTFRTCRAMLGEILSAYEFMDAECMKLVR-
Dog        176 VITAVSIQCPPKPQAVNVAFLGCPGFAEVLQTFSTCKGLLGEILSAYEFMDAECMWLVR-
Chicken    284 VITAVSILCPQKPKAVNLAFLGCQDFSRVQETFTTCRTMLGEILSAYEFMDEKCMELVE-
Frog       261 VITAVSILCPRKPKSVNLALLGCESFSRVLQVFTLCRDHLGEILSAYEFQDLESMRVVQ-
Zebrafish  275 VITAVSILCPRKPKAVNVAFLGCSSFQQLLETFQCCRGMLGEILSAFEFLDASCMNLLE-
Fruitfly   278 VVTKLSMLCPHSSRAVNVAFIGLNSFDDVLKTFVSAKRNLGEILSSCELIDERALNTALE
Yeast      232 VVTGVSIVAAAKPKALNAVFFGIENFDTVQKLFVKAKSELSEILSAFEFMDRGSIECTIE

                          E333L                                                 D375T
                          ▼                                                     ▼
Human      323 RHLHLASPVQ-ESPFYVLIETSGSNAGHDAEKLGHFLEHALGSGLVTDGTMATDQRKVKM
Chimp      323 RHLHLASPVQ-ESPFYVLIETSGSNAGHDAEKLGRFLEHVLGSGLVTDGTMATDQRKVKM
Rat        277 QHLHLTNPVQ-ESPFYVLVETSGSSAGHDAEKLTNVLEQVLNSGLVIDGTMATDQRKVQM
Mouse      337 QHLQLTNPVQ-ESPFYVLVETSGSSAGHDAEKLTNVLEQVLNSGLVTDGTMATDQRKVQM
Bovine     346 LHLGLSCPVQ-ESPFYVLIETAGSGPGHDAEKLGCFLEQVLDSGLVTDGTLGSDERRIKM
Dog        235 HHLHLTSPVQ-ESPFYVLIETSGSRAEHDAEKLNDFLEQALRSGLVTDGTVATDQMKLKA
Chicken    343 KHLGLSNPVR-GSPFYVLIETSGSNSTHDEEKLNSFLEQAMTSGLVTDGTVAVDDKKIKT
Frog       320 THLKLSNPLQ-ESAFYILIETAGSCEQHDEEKLNAFLAKALESGLVESGTVATDQGKIKS
Zebrafish  334 KHLKLTNPIT-ECPFYIVIETAGSNATHDEEKLHQFLEEVMTSSLVTDGTVATEATKIKA
Fruitfly   338 QFKFLNSPIS-GFPFYMLIETSGSNGDHDEEKINQFIGDGMERGEIQDGTVTGDPGKVQE
Yeast      292 YLKDLPFPLENQHNFYVLIETSGSNKRHDDEKLTAFLKDTTDSKLISEGMMAKDKKADFDR

                         A392G     V399M                     R419H                 A426T
                         ▼         ▼                         ▼                     ▼
Human      382 LWALRERITEALSRDGYVYKYDLSLPVERLYDIVTDLRARLGPH---------AKHVVGY
Chimp      382 LWALRERITEALSRDGYVYKYDLSLPVERLYDIVTDLRARLGPH---------AKHVVGY
Rat        336 LWALRERITEALSRDGYVFKYDLSLPVERLYDLVIDLRTRLGPR---------AKHVVGY
Mouse      396 LWALRERITEALSRDGYVFKYDLSLPVERLYDLVIDLRTRLGPR---------AKHVVGY
Bovine     405 LWALRERITEALSRDGYVYKYDLSLPLDRLYDLVGDLRARLGPS---------AKHVVGY
Dog        294 LWALRERISEALSRDGYVYKYDLSLPTDTLYDLVTDLRARLGSQ---------AKRVVGY
Chicken    402 LWSLRERITEALTRDGCVYKYDVSLPVGKLYDLVTDMRARLGQS---------AKNVVGY
Frog       379 LWALRERITEALAHDGYVYKYDLSIPVEKLYALVEETRARLGTS---------ARRVVGY
Zebrafish  393 LWSLRERVTEALTHEGYTYKYDISIPVEKIYDLVQDMRRHLGGM---------AKNVVGY
Fruitfly   397 IWKIREMVPLGLIEKSFCFKYDISLPLRDFYNIVDVMRERCGPL---------ATVVCGY
Yeast      352 LWTWRKSVPTACNSYGGMYKYDMSLQLKDLYSVSAAVTERLNAAGLIGDAPKPVVKSCGY

                                V444A
                G436V  N439D    | A446V   L453F                A474V  G477R
                ▼      ▼        ▼ ▼       ▼                    ▼      ▼
Human      433 GHLGDGNLHLNVTAEAFSPSLLAALEPHVYEWTAGQQGSVSAEHGVGFRKRDVLGYSKPP
Chimp      433 GHLGDGNLHLNVTAEAFSPSLLAALEPHVYEWTAGQQGSVSAEHGVGFRKRDVLGYSKPP
Rat        387 GHLGDGNLHLNVTAEAFSQELLGALEPYVYAWTAEQRGSVSAEHGLGFKKKNVLGYSKPP
Mouse      447 GHLGDGNLHLNVTAEAFSRELLGALEPYVYAWTAEQRGSVSAEHGLGFKKKDVLGYSKPP
Bovine     456 GHLGDGNLHLNVTSEAFSTSLLGALEPYVYEWTAGQRGSVSAEHGLGFKKKDVLGYSKPP
Dog        345 GHLGDGNLHLNVTSEDFSRSLLDALEPYVYEWTAGQRGSVSAEHGLGFKKKDALHYSKPP
Chicken    453 GHLGDGNLHLNITAESYSHSLLDAIEPFVYEWTARCNGSISAEHGLGFKKKQFIQYSKPN
Frog       430 GHLGDGNLHLNITSETYSESLLAALEPFVYQWTAGHSGSISAEHGLGFKKRDHIYYSKSS
Zebrafish  444 GHVGDGNLHLNITSPSKDFDLLAAIEPYVYEWTSQWKGSISAEHGLGLKKRNYIYYSKPS
Fruitfly   448 GHLGDSNLHLNVSCEEFNGEIYKRVEPFVYEYTSKLKGSISAEHGIGFLKKDYLHYSKDP
Yeast      412 GHVGDGNIHLNIAVREFTKQIEDLLEPFVYEYIASKKGSISAEHGIGFHKKGKLHYTRSD

Human      493 GALQLMQQLKALLDPKGILNPYKTLPSQA-
Chimp      493 GALQLMQQLKALLDPKGILNPYKTLPSQA-
Rat        447 VAVKLMQQLKAMLDPKGILNPYKTLPARA-
Mouse      507 VAVTLMQQLKAMLDPEGILNPYKTLPAQA-
Bovine     516 EALQLMRQLKALLDPKGILNPYKMLPTHA-
Dog        405 AALRLMQQLKALLDPKGILNPYKTLPAQA-
Chicken    513 EAVFLMQRFKAMLDPKGILNPYKTLPSSL-
Frog       490 EAVQLMQRLKKMLDPKGIMNPYKTIPQAD-
Zebrafish  504 EAVALMGSIKAMLDPKGILNPYKTLPDNIN
Fruitfly   508 VAIGYMREMKKLLDPNSILNPYKVLN----
Yeast      472 IEIRFMKDIKNHYDPNGILNPYKYI-----
```

Supp. Figure S2: Multiple sequence alignment of 11 homologues D-2-HGDH proteins.

The following D-2-HGDH sequences were used: Q8N465-*Homo sapiens* (Human); K6Z916-*Pan troglodytes* (Chimp); B2GV75-*Rattus norvegicus* (Rat); Q8CIM3-*Mus musculus* (Mouse); Q1JPD3-*Bos taurus* (Bovine); J9P2X0-*Canis lupus familiaris* (Dog); A0A1D5PIB0-*Gallus gallus* (Chicken); F7DLT4-*Xenopus tropicalis* (Frog); A1L258-*Danio rerio* (Zebrafish); Q7K511-*Drosophila melanogaster* (Fruitfly); P39976-*Saccharomyces cerevisiae* (Yeast). The D-2-HGDH sequences were retrieved from the https://www.uniprot.org, saved in FASTA format and aligned using the Clustal Omega program. The BOXSHADE program was used to visualise identical amino acids (in black) and functionally conserved amino acids (in grey). ”▼” indicates the position of the mutated amino acids characterized in this study.

Supp. Table S1: Primers used for site directed mutagenesis

**Primer**	**Sequence (5’ → 3’)**
D2HGDH_Ser109Trp_F	GGCCACGGACGTGGGAGGAGGTG
D2HGDH_Ser109Trp_R	CACCTCCTCCCACGTCCGTGGCC
D2HGDH_Leu124Pro_F	CACGAGAGGAACCCGGCCGTGAACCCA
D2HGDH_Leu124Pro_R	TGGGTTCACGGCCGGGTTCCTCTCGTG
D2HGDH_Ala125Thr_F	ACGAGAGGAACCTGACCGTGAACCCACAG
D2HGDH_Ala125Thr_R	CTGTGGGTTCACGGTCAGGTTCCTCTCGT
D2HGDH_Asn127Lys_F	ACCTGGCCGTGAAACCACAGGGGGG
D2HGDH_Asn127Lys_R	CCCCCCTGTGGTTTCACGGCCAGGT
D2HGDH_Gly131Val_F	GAACCCACAGGGGGTCAACACAGGCATGG
D2HGDH_Gly131Val_R	CCATGCCTGTGTTGACCCCCTGTGGGTTC
D2HGDH_Ile147Ser_F	TCCCCGTCTTTGACGAGATCAGCCTCTCCACTG
D2HGDH_Ile147Ser_R	CAGTGGAGAGGCTGATCTCGTCAAAGACGGGGA
D2HGDH_Met153Val_F	TCCACTGCCCGCGTGAACCGGGTCC
D2HGDH_Met153Val_R	GGACCCGGTTCACGCGGGCAGTGGA
D2HGDH_Met153Thr_F	CCACTGCCCGCACGAACCGGGTCCT
D2HGDH_Met153Thr_R	AGGACCCGGTTCGTGCGGGCAGTGG
D2HGDH_Gln169Pro_F	ATTCTGGTTTGCCCGGCGGGCTGCGTC
D2HGDH_Gln169Pro_R	GACGCAGCCCGCCGGGCAAACCAGAAT
D2HGDH_Ala170Glu_F	CTGGTTTGCCAGGAGGGCTGCGTCCTG
D2HGDH_Ala170Glu_R	CAGGACGCAGCCCTCCTGGCAAACCAG
D2HGDH_Cys172Tyr_F	GCCAGGCGGGCTACGTCCTGGAG
D2HGDH_Cys172Tyr_R	CTCCAGGACGTAGCCCGCCTGGC
D2HGDH_Pro189Leu_F	GGGACTTCATCATGCTGCTGGACTTAGGAGC
D2HGDH_Pro189Leu_R	GCTCCTAAGTCCAGCAGCATGATGAAGTCCC
D2HGDH_Ile200Thr_F	CAAGGGCAGCTGCCACACCGGGGGAAA
D2HGDH_Ile200Thr_R	TTTCCCCCGGTGTGGCAGCTGCCCTTG
D2HGDH_Ala205Val_F	CGGGGGAAACGTGGTAACCAACGCTGGAG
D2HGDH_Ala205Val_R	CTCCAGCGTTGGTTACCACGTTTCCCCCG
D2HGDH_Ala231Val_F	GAAGTGGTGCTGGTCGACGGCACTGTC
D2HGDH_Ala231Val_R	GACAGTGCCGTCGACCAGCACCACTTC
D2HGDH_Gly233Ser_F	GTGCTGGCCGACAGCACTGTCCTGG
D2HGDH_Gly233Ser_R	CCAGGACAGTGCTGTCGGCCAGCAC
D2HGDH_Cys272Arg_F	CGGTGTCCATCTTGCGTCCACCCAAGCCC
D2HGDH_Cys272Arg_R	GGGCTTGGGTGGACGCAAGATGGACACCG
D2HGDH_Glu311Lys_F	AGATCCTGTCTGCATTCAAGTTCATGGATGCTGTG
D2HGDH_Glu311Lys_R	CACAGCATCCATGAACTTGAATGCAGACAGGATCT

D2HGDH_Glu333Lys_F	GGCCAGCCCGGTGCAAAAGAGTCCGTTTTAC
D2HGDH_Glu333Lys_R	GTAAAACGGACTCTTTTGCACCGGGCTGGCC
D2HGDH_Asp375Tyr_F	GGACCATGGCCACCTACCAGAGGAAAGTC
D2HGDH_Asp375Tyr_R	GACTTTCCTCTGGTAGGTGGCCATGGTCC
D2HGDH_Ala392Gly_F	GGATCACAGAGGGGCTGAGCCGGGA
D2HGDH_Ala392Gly_R	TCCCGGCTCAGCCCCTCTGTGATCC
D2HGDH_Val399Met_F	AGCCGGGATGGCTACATGTACAAGTACGACC
D2HGDH_Val399Met_F	GGTCGTACTTGTACATGTAGCCATCCCGGCT
D2HGDH_Arg419His_F	GACTGACCTGCACGCCCGCCTCG
D2HGDH_Arg419His_R	CGAGGCGGGCGTGCAGGTCAGTC
D2HGDH_Ala426Thr_F	CTCGGCCCGCACACCAAGCACGTGG
D2HGDH_Ala426Thr_R	CCACGTGCTTGGTGTGCGGGCCGAG
D2HGDH_Gly436Val_F	GGCTATGGCCACCTTGTAGATGGTAACCTGCAC
D2HGDH_Gly436Val_R	GTGCAGGTTACCATCTACAAGGTGGCCATAGCC
D2HGDH_Asn439Asp_F	TGGCCACCTTGGAGATGGTGACCTGCACCT
D2HGDH_Asn439Asp_R	AGGTGCAGGTCACCATCTCCAAGGTGGCCA
D2HGDH_Val444Ala_F	ACCTGCACCTCAATGCGACGGCGGAGG
D2HGDH_Val444Ala_R	CCTCCGCCGTCGCATTGAGGTGCAGGT
D2HGDH_Ala446Val_F	CCTCAATGTGACGGTGGAGGCCTTCAGCC
D2HGDH_Ala446Val_R	GGCTGAAGGCCTCCACCGTCACATTGAGG
D2HGDH_Leu453Phe_F	TTCAGCCCCTCGTTCCTGGCTGCCC
D2HGDH_Leu453Phe_R	GGGCAGCCAGGAACGAGGGGCTGAA
D2HGDH_Ala474Val_F	GCAGCGTCAGCGTGGAGCACGGAGT
D2HGDH_Ala474Val_R	ACTCCGTGCTCCACGCTGACGCTGC
D2HGDH_Gly477Arg_F	CAGCGCGGAGCACAGAGTGGGCTTCAG
D2HGDH_Gly477Arg_R	CTGAAGCCCACTCTGTGCTCCGCGCTG

F=Forward; R=Reverse; Underlined=mismatched nucleotide

Supp. Table S2: PolyPhen-2, SIFT and MutationTaster *in silico* pathogenicity predictions of the 31 studied missense variants

Variant No.	Exon	DNA change	Protein change	PolyPhen-2	SIFT	Mutation Taster	Residual activity(%)
1[b]	3	c.326C>G	p.Ser109Trp	probably damaging (score 0.989)	deleterious (score 0)	polymorphism (p value 0.972)	3
2[a]	4	c.371T>C	p.Leu124Pro	probably damaging (score 1)	deleterious (score 0)	disease causing (p value 1)	0
3[a]	4	c.373G>A	p.Ala125Thr	probably damaging (score 1)	deleterious (score 0)	disease causing (p value 1)	6
4[c]	4	c.381C>A	p.Asn127Lys	benign (score 0.445)	tolerated (score 0.27)	disease causing (p value 0.987)	0
5[a]	4	c.392G>T	p.Gly131Val	probably damaging (score 1)	deleterious (score 0)	disease causing (p value 1)	0
6[a]	4	c.440T>G	p.Ile147Ser	probably damaging (score 0.996)	deleterious (score 0)	disease causing (p value 1)	0
7[a]	4	c.457A>G	p.Met153Val	probably damaging (score 0.989)	deleterious (score 0.04)	disease causing (p value 1)	2
8[a]	4	c.458T>C	p.Met153Thr	probably damaging (score 0.999)	deleterious (score 0.01)	disease causing (p value 1)	0
9[c]	5	c.506A>C	p.Gln169Pro	benign (score 0.015)	tolerated (score 0.08)	disease causing (p value 1)	0
10[a]	5	c.509C>A	p.Ala170Glu	probably damaging (score 0.998)	deleterious (score 0.01)	disease causing (p value 1)	0
11[c]	5	c.515G>A	p.Cys172Tyr	possibly damaging (score 0.875)	tolerated (score 0.09)	disease causing (p value 1)	2
12[a]	5	c.566C>T	p.Pro189Leu	probably damaging (score 1)	deleterious (score 0)	disease causing (p value 1)	2
13[a]	5	c.599T>C	p.Ile200Thr	probably damaging (score 0.972)	deleterious (score 0.01)	disease causing (p value 1)	1

14[a]	5	c.614C>T	p.Ala205Val	probably damaging (score 0.972)	deleterious (score 0.02)	disease causing (p value 1)	40
15[c]	6	c.692C>T	p.Ala231Val	benign (score 0.202)	tolerated (score 0.12)	disease causing (p value 1)	45
16[a]	6	c.697G>A	p.Gly233Ser	probably damaging (score 0.999)	deleterious (score 0)	disease causing (p value 1)	5
17[a]	6	c.814T>C	p.Cys272Arg	probably damaging (score 0.992)	deleterious (score 0.01)	disease causing (p value 1)	0
18[a]	7	c.931G>A	p.Glu311Lys	probably damaging (score 1)	deleterious (score 0)	disease causing (p value 1)	0
19[b,*]	7	c.997G>A	p.Glu333Lys	benign (score 0.010)	tolerated (score 0.28)	disease causing (p value 1)	57
20[a]	8	c.1123G>T	p.Asp375Tyr	probably damaging (score 1)	deleterious (score 0.01)	disease causing (p value 1)	17
21[b]	9	c.1175C>G	p.Ala392Gly	possibly damaging (score 0.555)	tolerated (score 0.19)	disease causing (p value 1)	59
22[b]	9	c.1195G>A	p.Val399Met	probably damaging (score 0.995)	tolerated (score 0.17)	disease causing (p value 1)	25
23[a]	9	c.1256G>A	p.Arg419His	probably damaging (score 1)	deleterious (score 0.02)	disease causing (p value 1)	37
24[c]	9	c.1276G>A	p.Ala426Thr	benign (score 0.410)	deleterious (score 0.03)	disease causing (p value 1)	94
25[d]	10	c.1307G>T	p.Gly436Val	probably damaging (score 0.999)	deleterious (score 0)	disease causing (p value 1)	87
26[a]	10	c.1315A>G	p.Asn439Asp	probably damaging (score 0.974)	deleterious (score 0)	disease causing (p value 1)	36
27[a]	10	c.1331T>C	p.Val444Ala	possibly damaging (score 0.698)	deleterious (score 0.01)	disease causing (p value 1)	23
28[a]	10	c.1337C>T	p.Ala446Val	benign (score 0.023)	tolerated (score 0.47)	polymorphism (p value 1)	67
29[d]	10	c.1357C>T	p.Leu453Phe	possibly damaging (score 0.559)	deleterious (score 0.03)	disease causing (p value 0.996)	68
30[a]	10	c.1421C>T	p.Ala474Val	probably damaging (score 1)	deleterious (score 0)	disease causing (p value 1)	1

31[a]	10	c.1429G>A	p.Gly477Arg	probably damaging (score 1)	deleterious (score 0)	disease causing (p value 1)	0

PolyPhen-2, SIFT and MutationTaster *in silico* predictions were obtained via Alamut Visual version 2.9. PolyPhen-2 score rages from 0.0 (tolerated) to 1.0 (deleterious). The SIFT score ranges from 0.0 (deleterious) to 1.0 (tolerated) with a threshold of 0.05. The “p” value generated by the MutationTaster represents the probability of the prediction, i.e. a value close to 1 indicates a higher reliability of the prediction. The results were compared within each other and also against the functional studies of the missense variants in HEK cells. The estimated pathogenic effect is not always consistent within the used predictions sites or with the actual measured activities (predictions highlighted in red). For 20 out of 31 variants (64.5%; variant no. marked with [a]) all three software tools were unanimous in assigning pathogenicity predictions and were also in agreement with the results of the functional studies in HEK cells. For four variants (13%; variant no. marked with [b]), consistent pathogenicity prediction, in line with the functional data, was provided by two out of three software tools. For five variants (16%; variant no. marked with [c]) only one prediction tool was in agreement with the functional data. Two variants (6.5%; variant no. marked with [d]) were predicted pathogenic by all tools, but showed very high residual activity in our overexpression system. The potential effect of the variants on mRNA splicing was also evaluated using *in silico* splice tools: MaxEntScan, NNsplice and Human Splicing Finder (Alamut Visual software, version 2.9).

*Only for c.997G>A; (p.Glu333Lys) variant the predicted splicing events appeared to be significant: predicted change at donor site 1 bps downstream: -71.2% (MaxEntScan: -100.0%; NNSPLICE: -99.7%; Human Splicing Finder: -13.9%).
